# How Temperature, Pressure, and Salt Concentration Affect Correlations in LiTFSI/EMIM-TFSI Electrolytes: A Molecular Dynamics Study

**DOI:** 10.1021/acs.jpcb.1c07782

**Published:** 2021-10-28

**Authors:** Piotr Kubisiak, Piotr Wróbel, Andrzej Eilmes

**Affiliations:** Faculty of Chemistry, Jagiellonian University, Gronostajowa 2, 30-387 Kraków, Poland

## Abstract

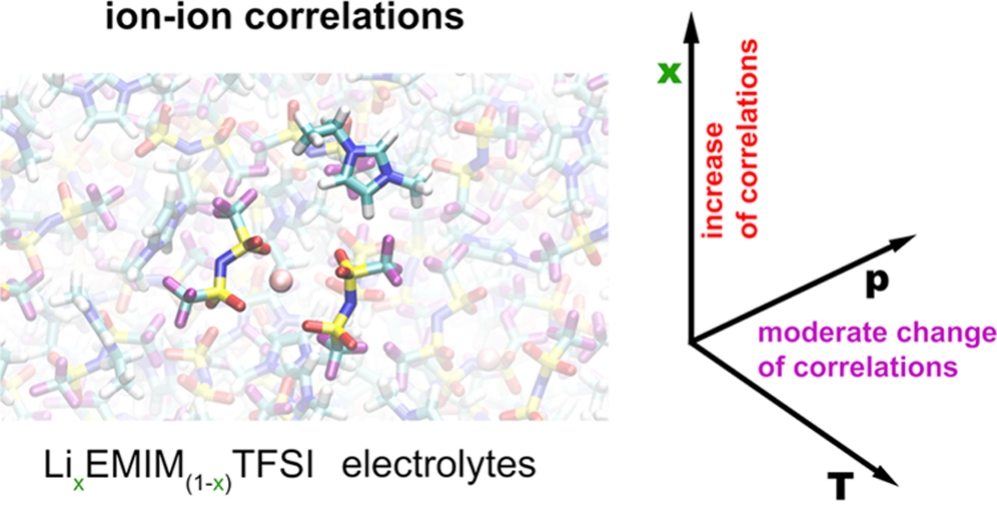

Classical polarizable molecular dynamics simulations have been performed for LiTFSI solutions in the EMIM-TFSI ionic liquid. Different temperature or pressure values and salt concentrations have been examined. The structure and dynamics of the solvation shell of Li^+^ cations, diffusion coefficients of ions, conductivities of the electrolytes, and correlations between motions of ions have been analyzed. The results indicated that regardless of the conditions, significant correlations are present in all systems. The degree of correlations depends mainly on the salt fraction in the electrolyte and is much less affected by temperature and pressure changes. A positive correlation between motions of Li^+^ cations and TFSI anions, leading to the occurrence of negative Li^+^ transference numbers, exists for all conditions, although temperature and pressure changes affect the speed of anion exchange in Li^+^ solvation shells.

## 1 Introduction

One of the key technological challenges of modern society is to meet the increasing demand for environmentally friendly, safe, cheap, and easy-to-manufacture energy storage devices. Since the first commercial Li-ion batteries became available in the early 1990s of the 20th century, they found a range of successful applications from portable devices for mobile electronics and electric vehicles to large stationary energy storage systems. A large effort is therefore invested in developing new, effective solutions, not only limited to Li-based devices^1−3^ but also including alternative chemistries, such as Na-ion batteries.^4−6^

The ion-conducting electrolyte, typically a metal salt solution in a liquid or a polymer matrix, is an important component of metal-ion batteries. In search of optimized electrolytes, ionic liquids (ILs) gained significant attention,^7−14^ owing to their several advantages, such as stability and nonvolatility. Experimental works and computational modeling cover a range of temperatures of possible operating conditions between the melting and boiling points of the IL.^15−20^ Studies on pressure effects on ILs focus mainly on structural and conformational changes or glass transitions,^21−31^ but there are also several works investigating transport properties—diffusion coefficients or conductivity.^21,26,32−35^

Cross-correlations between ion motions play an important role in charge transport and conductivity of an electrolyte. Analysis of correlations and their effect on transference numbers or ion transport was reported for Li salt/glyme solutions,^36−40^ IL/molecular solvent blends,^41,42^ ILs, or salt solutions in ILs.^43−47^ Recently, negative effective transference numbers of Li^+^ cations were determined for LiTFSI solutions in EMIM-TFSI IL^48^ and attributed to Li^+^-anion correlations. Subsequent molecular dynamics (MD) studies on NaFSI solutions in EMIM-FSI IL,^49^ a series of electrolytes based on lithium salts dissolved in EMIM ILs with different anions,^50^ or MeTFSI (Me = Li, Na) solutions in EMIM-TFSI^51^ demonstrated that the effect of negative transference numbers of metal cations emerges from strong correlations in ILs and is expected to appear in certain range of salt concentrations. The effect may be reduced by the addition of a molecular chelating agent to the IL electrolyte.^52,53^

Changes in the temperature and pressure affect the density of the electrolyte, interactions between ions, lifetime of ion aggregates, and mobility of ions. It is therefore natural to ask the question whether increase of temperature or pressure can change the degree of correlations in an IL and the ion transference numbers. In this work, we wanted to investigate this problem by MD simulations. As a model system, we used a typical IL-based Li-conducting electrolyte, LiTFSI in the EMIM-TFSI liquid. We performed simulations for several values of temperature and pressure as well as for increasing salt load to compare the effects of *pT* changes with those observed for the increase of Li^+^ concentration.

## 2 Computational Methods

We studied the Li_*x*_EMIM_(1–*x*)_TFSI electrolytes at two salt mole fractions, *x* = 0.1 and 0.2, and the neat EMIM-TFSI IL (*x* = 0). Initial structures were prepared using the Packmol program.^54^ The systems consisted of 142 EMIM-TFSI ion pairs (neat IL), 15 LiTFSI ion pairs in 135 pairs of IL ions (*x* = 0.1), and 34 LiTFSI in 136 EMIM-TFSI pairs (*x* = 0.2).

MD simulations were performed in the NAMD v. 2.12^55^ simulation package. Parameterization of the polarizable force field was the same as in our recent work.^51^ The EMIM-TFSI liquid was based on optimized potentials for liquid simulations (OPLS) parameterization^56^ with bonded parameters taken from the Lopes/Pádua field^57^ and nonbonded parameters from Köddermann’s work.^58^ Nonbonded parameters for Li^+^ were taken from ref (59). Polarization was introduced via Drude particles.^60^ Atomic polarizabilities and charges were adapted from the APPLE&P polarizable force field for liquids and electrolytes.^61^ For more details of parameterization, the Reader is referred to ref (51).

Initial NAMD simulations were performed in the *NpT* ensemble with Langevin dynamics and the modified Nosé–Hoover Langevin barostat.^62,63^ A time step of 0.5 fs was used to integrate equations of motion. Periodic boundary conditions were applied to the system, and electrostatic interactions were taken into account via the particle mesh Ewald algorithm.^64^ A cutoff value of 12 Å was used for the electrostatic and van der Waals interactions. To study the effect of pressure, three different pressures, *p* = 1, 100, or 1000 atm, were applied to systems at *T* = 300 K. Two other series of systems were simulated under *p* = 1 atm at elevated temperatures, *T* = 400 and 450 K. For each of the five *p* and *T* pairs, we simulated three salt fractions *x*, yielding in total 15 combinations of *x*, *p*, and *T* values. To average the results, 10 different system realizations were used for each *x*, *p*, and *T* combination. When the density of the system became constant after about 10–30 ns of *NpT* simulations, we performed 150 ns long production runs in the *NVT* ensemble using the box sizes corresponding to the average densities obtained at the *NpT* stage. Box sizes and information about pressure fluctuations observed in MD runs are presented in the Supporting Information (Table S1 and Figures S1 and S2). The last 120 ns of each trajectory were used to estimate the diffusion coefficients and conductivity; then, the results were averaged over 10 systems.

## 3 Results and Discussion

### 3.1 Structure

Average densities of all simulated systems are collected in Table 1. The value of 1.513 g/cm^3^ calculated at 300 K, 1 atm agrees well with the experimental result, 1.517 g/cm^3^.^65^ Increase of pressure from 1 to 100 atm results in a small (less than 1%) density increase. A much larger change is observed at 1000 atm: at this pressure, densities of electrolytes are 7–8% larger than under normal conditions. As expected, at increased temperatures, the density of the solution decreases; the changes amount to 9 and 13% for 400 and 450 K, respectively. For the neat IL, the experimental data of ref (65), extrapolated to 400 and 450 K, yield the densities about 0.04–0.05 g/cm^3^ larger than our simulated values. At 300 K, increasing the LiTFSI content increases the density, and the change is the largest at 1000 atm (increase by 0.03 g/cm^3^ from neat IL to the *x* = 0.2 electrolyte). However, the calculated dependence of the density on salt concentration is weaker than that observed experimentally: at 300 K, 1 atm, the interpolation/extrapolation of the data for Li_*x*_EMIM_(1–*x*)_TFSI electrolytes from ref (66) yields the densities 0.02 and 0.04 g/cm^3^ larger than the values obtained for *x* = 0.1 and 0.2, respectively. At high temperatures, the salt concentration has a very small impact on the density in MD simulations. At 400 K, densities of all electrolytes are practically the same within 0.001 g/cm^3^ and at 450 K, the trend is even reversed: increase of salt fraction by 0.1 results in the density decrease by 0.001 g/cm^3^.

**Table 1:** Average Densities of Simulated Electrolytes with Standard Deviations (g/cm^3^)

	300 K, 1 atm	300 K, 100 atm	300 K, 1000 atm	400 K, 1 atm	450 K, 1 atm
*x* = 0	1.513 ± 0.001	1.526 ± 0.001	1.612 ± 0.001	1.377 ± 0.001	1.313 ± 0.001
*x* = 0.1	1.518 ± 0.002	1.533 ± 0.001	1.627 ± 0.001	1.378 ± 0.001	1.312 ± 0.001
*x* = 0.2	1.522 ± 0.002	1.539 ± 0.002	1.643 ± 0.002	1.378 ± 0.001	1.311 ± 0.002

**Figure 1:**
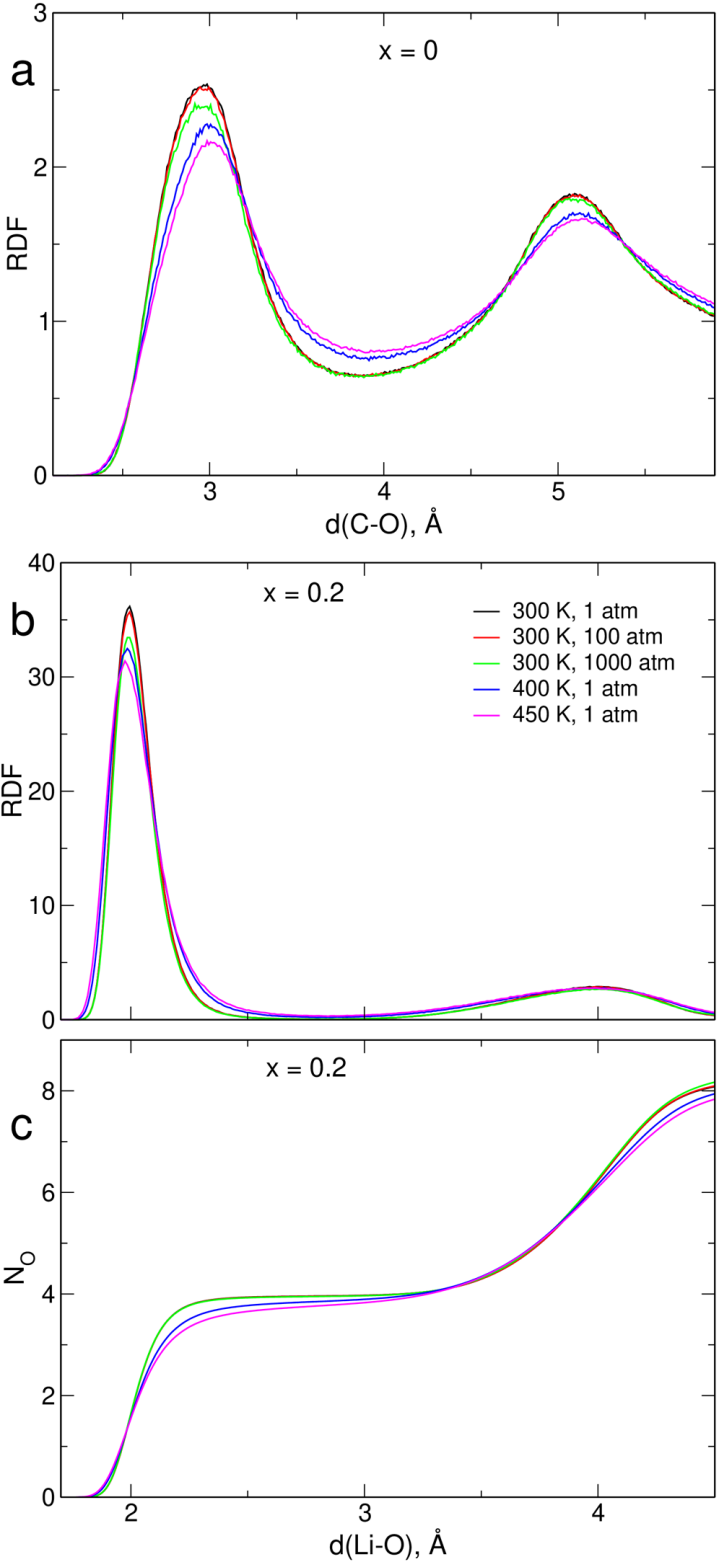
C–O RDF in neat IL (a); Li–O RDF (b); and integrated Li–O RDF in the *x* = 0.2 electrolyte (c).

Sample plots of radial distribution functions (RDFs) for C–O and Li–O pairs for the selected electrolyte concentrations are shown in Figure 1; here, C denotes the carbon atom located between two nitrogen atoms in the 5-membered ring of EMIM cation. We also presented the integrated Li–O RDF
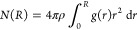
1where *g*(*r*) is the Li–O RDF and ρ is the average density of O atoms.

The position of the first maximum at about 3 Å in the C–O RDF is almost independent of pressure; only a slight shift to lower distances is noticeable for the 1000 atm data. Increase of temperature from 300 K to 400 or 450 K shifts the maximum by about 0.05 Å toward longer distances. Similar changes are observed for the second maximum at 5.1 Å. The first maximum at 2 Å in the Li–O RDF of the *x* = 0.2 system becomes a little wider at higher temperatures. The maximum in Li–O_TFSI_ RDF is typically reported at this distance for LiTFSI solutions in ILs with TFSI anions.^8,10^ Its position in Figure 1 does not depend on pressure but shifts to lower distances at higher *T*, as observed, e.g., for the Li–O_TFSI_ maximum in the LiTFSI/IL/poly(ethylene oxide) electrolyte.^67^ This effect is, however, very small as seen in the plot of integrated Li–O RDF (running Li coordination number (CN))—at 2 Å, the average number of oxygen atoms is the same for all systems within the resolution of the plot. Between 2 and 3 Å, integrated RDFs for different pressures at 300 K are the same; on the other hand, lower coordination numbers (CNs) are observed for systems simulated at 400 and 450 K. The Li^+^–O distances depend on interactions between atoms, balancing the electrostatic attraction and the van der Waals repulsion, and the pressure increase is not able to further compress the coordination shell of Li^+^, which is therefore quite insensitive to pressure up to 1000 atm. The higher temperature loosens the coordination, and thus the running CNs for 400 and 450 K are smaller. These observations are confirmed by the values of Li^+^ CN calculated at 3 Å, as shown in Table 2. Increase of temperature to 400 or 450 K decreases the CN by 0.08 and 0.14, respectively. At higher distances, the integrated RDF does not depend on the local coordination structure but reflects the average density of the system. Accordingly, above 4 Å, the sequence of curves in Figure 1 corresponds to the density of the electrolyte; that is, the average CNs decrease for lower pressures and/or higher temperatures.

**Table 2:** Average Coordination Numbers of Li^+^ Cations

	300 K, 1 atm	300 K, 100 atm	300 K, 1000 atm	400 K, 1 atm	450 K, 1 atm
*x* = 0.1	3.972	3.971	3.953	3.894	3.830
*x* = 0.2	3.973	3.970	3.966	3.897	3.831

**Figure 2:**
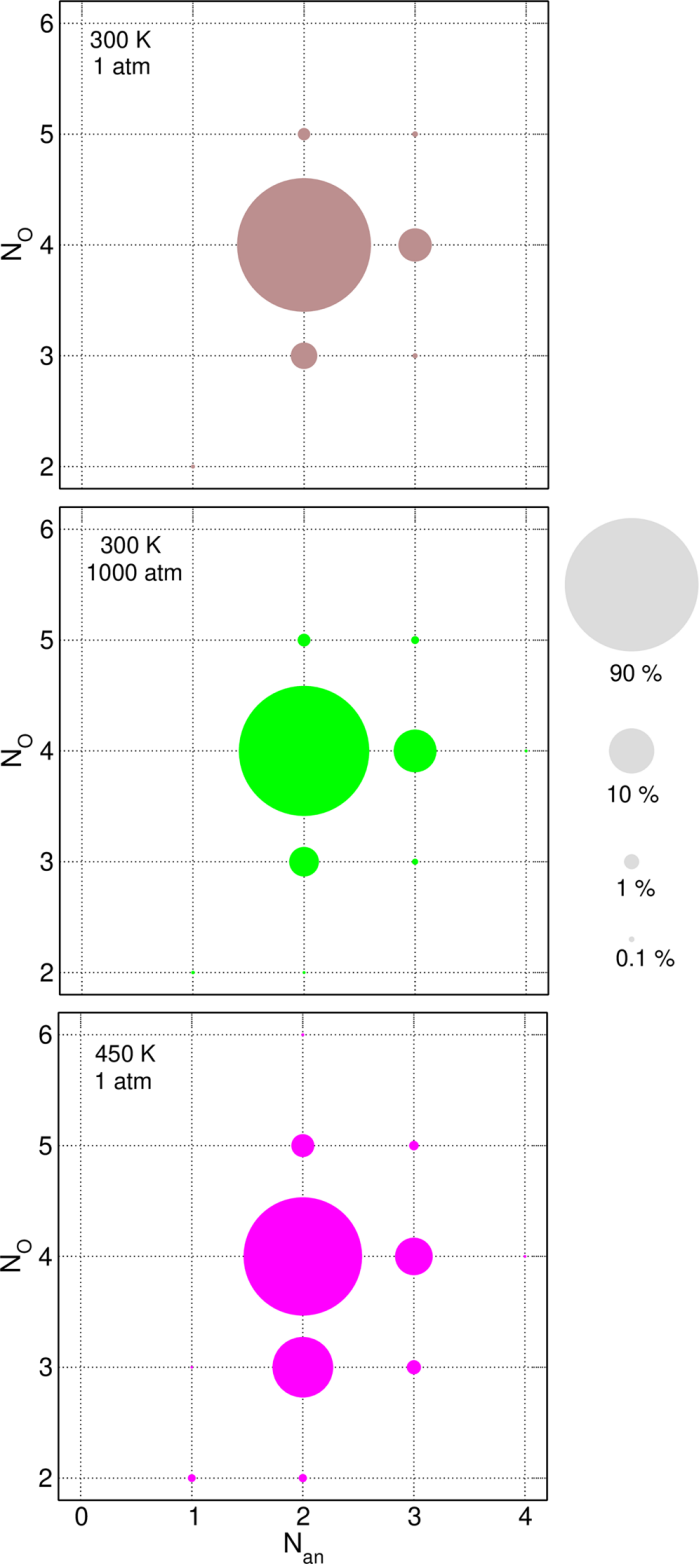
Abundance of different combinations of the number of anions *N*_an_ and the number of oxygen atoms *N*_O_ coordinating Li^+^ ions for the *x* = 0.2 electrolyte. Areas of circles are proportional to the abundance.

From Table 2 and Figure 1, one may conclude that the lithium ion is coordinated to about four oxygen atoms. To gain more information on the structure of the coordination shell, we displayed in Figure 2 the relative abundance of different numbers of coordinating anions *N*_an_ and oxygen atoms *N*_O_ in the *x* = 0.2 electrolyte. Areas of circles in Figure 2 are normalized in such a way that the total area in each panel corresponds to 100%. In all cases, the most probable is the coordination by four O atoms from two different anions (90.4% at 300 K, 1 atm; 85.7% at 300 K, 1000 atm, and 70.7% at 450 K, 1 atm). The cases of coordination by four O atoms from three anions or by three oxygens from two anions are less probable (5.3 and 3.3%, respectively, at 300 K, 1 atm). The former configuration becomes more abundant at a higher pressure (9.0% at 1000 atm), while the latter is favored at an increased temperature (18.2% at 450 K). The high temperature also increases the probabilities (although still very small) of other *N*_an_ and *N*_O_ combinations. We can conclude that regardless of the conditions, most Li^+^ cations are coordinated by two anions providing four or (less frequently) three coordinating O atoms. The bidentate environment of Li-TFSI pairs is the most probable. In the *x* = 0.2 electrolyte at 300 K, 1 atm, about 93% of anions interacting directly with Li^+^ ions are coordinated as bidentate. At the same temperature at 1000 atm, the probability of bidentate coordination decreases slightly to 89%. Larger changes are observed with increasing temperature: the amount of bidentate coordination is 87 and 83% at 400 and 450 K, respectively. Values calculated for the *x* = 0.1 system are systematically lower by 1–2%.

### 3.2 Dynamics

As shown in the preceding section, changes of pressure and temperature have a rather limited impact on the local structure in LiTFSI/EMIM-TFSI solutions. However, they have a greater effect on the dynamics and transport properties of the electrolyte.

In the research of metal ion-conducting electrolytes, the most interesting are the Me^+^–solvent interactions. To get some insight into the timescale of anion exchange in the solvation shells of lithium cations, we computed the O atom residence time autocorrelation functions (ACFs)
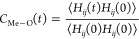
2where *H*_*ij*_(*t*) = 1 if the distance between the *i*th Li^+^ ion and the *j*th O atom is smaller than the threshold value of 3 Å, or *H*_*ij*_(*t*) = 0 otherwise. Likewise, anion residence time ACFs *C*_Me-an_(*t*) were defined, assuming that *H*_*ij*_(*t*) = 1 if the *j*th TFSI anion is coordinated to the *i*th Li^+^; that is, any O atom from the anion is within the threshold distance to the cation. As a single qualitative measure, we calculated anion residence times τ_an_ and oxygen atom residence times τ_O_ from the fit of the stretched exponential function exp[−(*t*/τ)^α^] to the residence time ACF.

**Figure 3:**
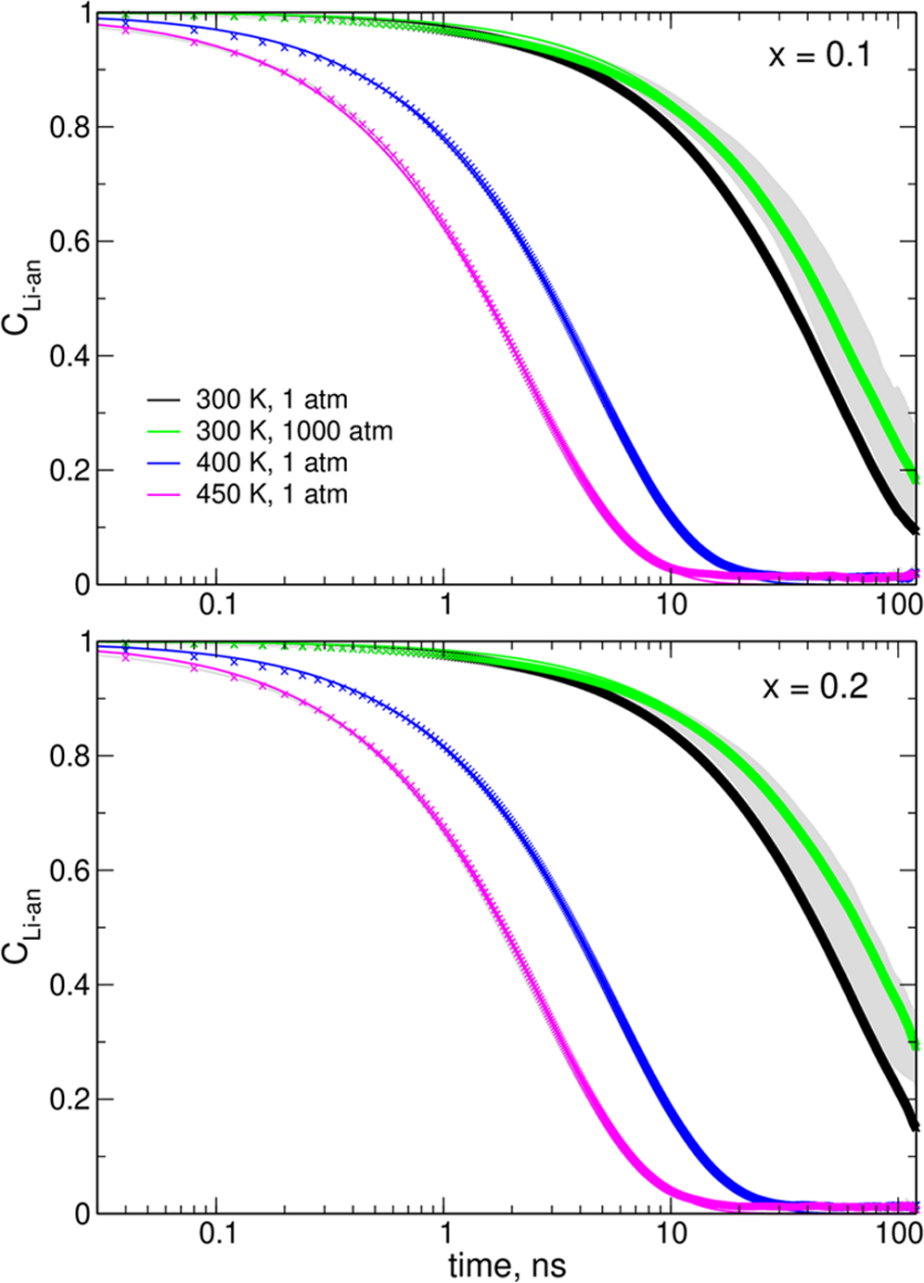
Li-anion residence time autocorrelation functions (symbols) and exponential fits to the data (lines). Uncertainties of calculated values are shown as gray area for the 300 K, 1000 atm and the 450 K, 1 atm data.

Plots of *C*_Me-an_(*t*) ACFs with fitting curves are shown in Figure 3. Uncertainties of the averages calculated for 10 realizations of the system are presented as the gap between the lowest and the highest values; for clarity, we have shown the gaps for only two data sets. The uncertainties are noticeable at 300 K, whereas at 450 K they are contained within the symbol size in the plot. The calculated residence times are collected in Tables 3 and 4.

**Table 3:** Estimated Oxygen Atom Residence Times, τ_O_ (ns)

	300 K, 1 atm	300 K, 100 atm	300 K, 1000 atm	400 K, 1 atm	450 K, 1 atm
*x* = 0.1	17.0	18.2	22.6	1.38	0.65
*x* = 0.2	21.4	21.6	31.2	1.67	0.75

**Table 4:** Estimated Anion Residence Times, τ_an_ (ns)

	300 K, 1 atm	300 K, 100 atm	300 K, 1000 atm	400 K, 1 atm	450 K, 1 atm
*x* = 0.1	48.1	51.4	68.4	4.5	2.3
*x* = 0.2	63.9	64.8	99.5	5.6	2.7

Breaking a single Li–O interaction in most cases does not imply breaking Li-TFSI association (because usually there are more Li–O bonds to the same anion); therefore, the anion residence times τ_an_ are about 3 times larger than the atom residence times τ_O_. Increase of pressure from 1 to 100 atm leads to only small increase of residence times; larger effect is observed at 1000 atm, especially for τ_an_ and/or in a more concentrated electrolyte *x* = 0.2. Change of temperature has a much stronger effect and the residence times at 450 K are 20–30 times smaller than those at 300 K.

**Figure 4:**
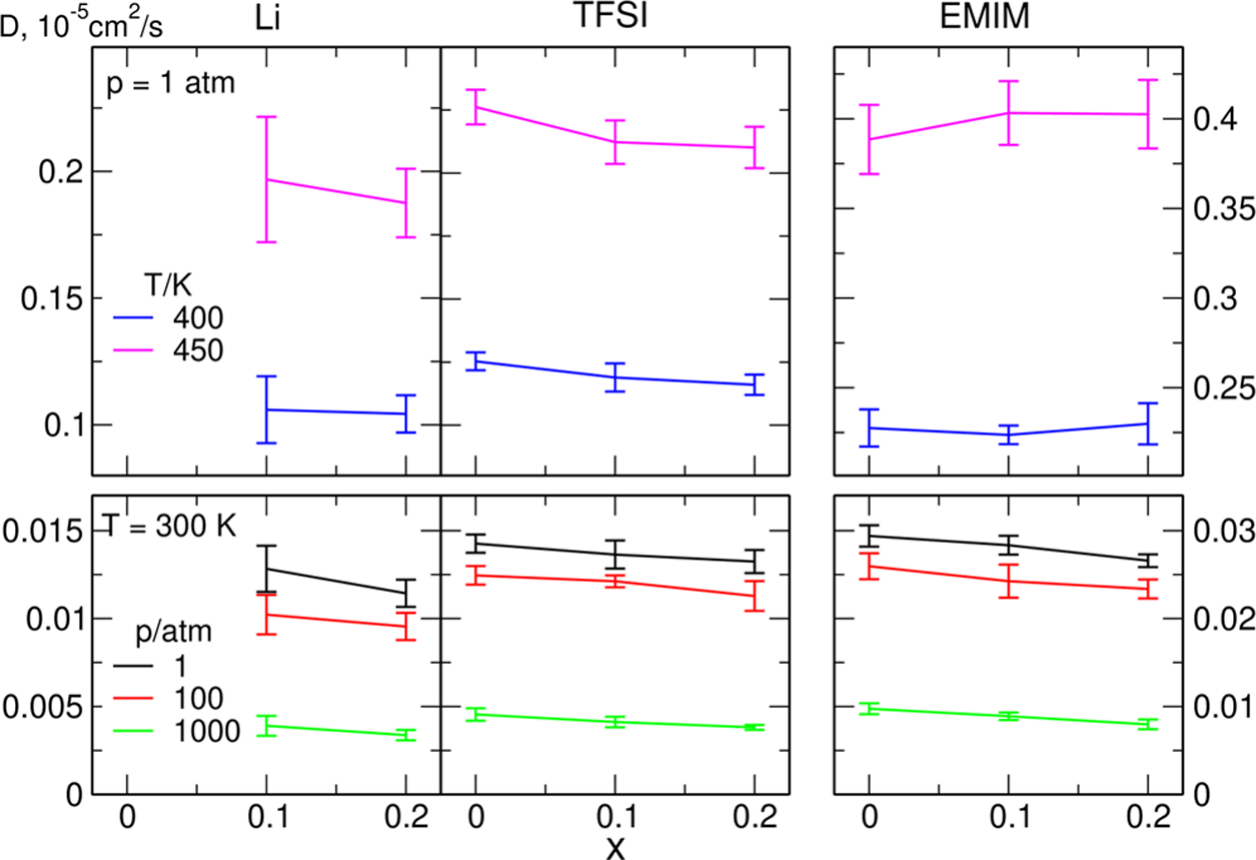
Calculated diffusion coefficients of ions. Note the scale difference between the panels.

Diffusion coefficients of ions were estimated from the displacements recorded in MD trajectories: the diffusion coefficient *D*_*i*_ of ion *i* was calculated from the slope of the time dependence of its mean square displacement (MSD)
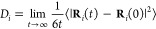
3Here, **R**_*i*_(*t*) is the position of the *i*th ion at time *t* and the brackets ⟨ ⟩ denote the ensemble average. The MSDs were averaged over all possible choices of time intervals Δ*t* within the last 120 ns of the MD trajectory; then, the data within the range of 10–30 ns were used to calculate the slope of MSD(*t*) dependence. Estimated diffusion coefficients are displayed in Figure 4; error bars mark the standard deviation of the results obtained for 10 different MD runs.

Increase of temperature to 400–450 K increases the diffusivity of ions by an order of magnitude. As readily seen, increase of pressure reduces the diffusion coefficients. The values obtained at 100 atm are only 10–20% lower than those calculated at 1 atm, but at 1000 atm, they decrease to less than half of the value estimated at a normal pressure. For neat EMIM-TFSI at 300 K, the increase of pressure from 1 to 1000 atm reduces diffusion coefficients by about 3 times for EMIM and 3.1 times for TFSI. Experimental data from ref (26) exhibit a stronger pressure dependence: *D*_EMIM_ decreases about 5 times and *D*_TFSI_ by an order of magnitude; the larger sensitivity of anions to pressure changes is the main difference between measurements and our estimates. It should be noted that parameterization of the FF for simulations at a high pressure is particularly difficult—the repulsive part of the van der Waals potential becomes increasingly important when the average distances between molecules/atoms decrease. The FF is typically developed for the densities at standard conditions; therefore, the short range part of the potential may not be optimal at an increased pressure.

Regardless of the conditions, the diffusion coefficients of Li^+^ cations and TFSI anions are similar, with *D*_Li_ only a little smaller than *D*_TFSI_. In all cases, EMIM cations are the most mobile, with *D*_EMIM_ about 2 times larger than the diffusion coefficients of other ions. Increase of the salt load leads to the decrease of the diffusivity of Li^+^ and TFSI ions as well as EMIM cations at *T* = 300 K. At higher temperatures, there is no clear trend in the changes of *D*_EMIM_ with increasing salt concentration. Our results obtained at 300 K, 1 atm can be compared to data from ref (48). MD simulations predicted slower decrease of diffusion coefficients upon increasing the salt concentration and the estimated values are lower than those measured. However, our values of 2.8 × 10^–7^, 1.4 × 10^–7^, and 1.3 × 10^–7^ cm^2^/s for *D*_EMIM_, *D*_TFSI_, and *D*_Li_ in the *x* = 0.1 electrolyte, respectively, compare well with the values 3.6 × 10^–7^, 1.8 × 10^–7^, and 1.0 × 10^–7^ cm^2^/s reported^48^ at 298 K for 0.5 mol/dm^3^ concentration, corresponding roughly to *x* = 0.12.

Conductivities of the studied systems were calculated from the Einstein relation as
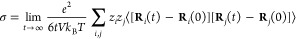
4In the above formula, *t* is the time, *V* is the volume of the simulation box, *k*_B_ is the Boltzmann constant, *T* is the temperature, *e* is the elementary charge, *z*_*i*_ and *z*_*j*_ are the charges of ions *i* and *j*, **R**_*i*_(*t*) is the position of the *i*th ion at time *t*, and the brackets ⟨ ⟩ denote the ensemble average. To compute the conductivity, we applied the same time range as used to calculate the diffusion coefficients. To improve the estimates of conductivity, we averaged the results over 10 system realizations, applying the procedure proposed in a recent paper,^68^ based on the correlation between the value of σ and the exponent α in the relation MSD ∼ *t*^α^.

**Figure 5:**
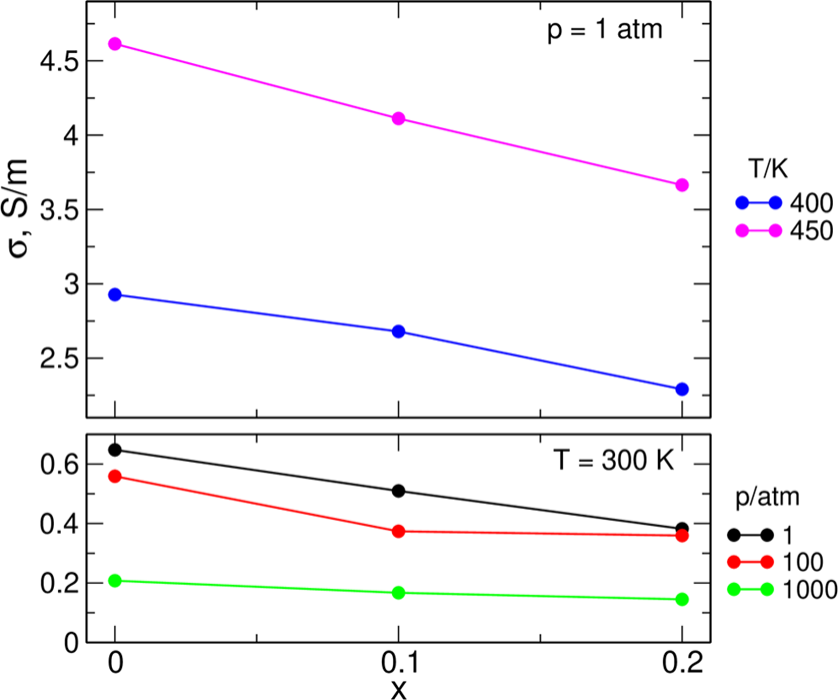
Estimated conductivities of the Li_*x*_EMIM_(1–*x*)_TFSI electrolytes.

Calculated conductivities for all systems are displayed in Figure 5. For clarity, we did not mark the error bars on the plot. The standard deviations of the data sets consisting of 10 samples reach about 50% of the average value. This is not surprising because such behavior was observed in a model study on conductivity estimates from MD simulations.^68^ Therefore, the use of standard deviation of the ensemble would likely overestimate the uncertainty related to the length of the trajectory and the time window used to calculate the slopes of MSD lines. Instead, we estimated the relative errors as 10–20% of the σ value from the analysis of stability of averaged values against the changes of time intervals or the number of samples used.

The trends in Figure 5 are similar to those presented earlier for diffusion coefficients. Increase of the pressure decreases the σ values, whereas the increase of the temperature from 300 K to 400 or 450 K largely enhances the conductivity of the electrolyte. At 300 K, the conductivity decreases 3 times upon the increase in the pressure from 1 to 1000 atm. In ref (35), a larger change (about an order of magnitude) was reported for BMIM-TFSI, but the overall effects are comparable (and the experimental dependence is weaker for other cations). The increasing amount of LiTFSI dissolved in the IL results in the decrease in conductivity. Such a trend is typically observed for salt solutions in ILs. Conductivity values obtained from impedance spectroscopy in ref (48) are 0.8 S/m for neat EMIM-TFSI and 0.55 S/m for LiTFSI at *x* = 0.12. Our values of 0.64 and 0.51 S/m calculated at 300 K, 1 atm for *x* = 0 and 0.1, respectively, agree well with experimental results. From the observation that the conductivity generally follows the changes of diffusion coefficients, one may presume that the degree of correlations between ion motions is not affected significantly by temperature or pressure changes. On the other hand, conductivity decreases faster than the averaged diffusion coefficients when the salt content in the electrolyte is increased, suggesting an increase of destructive correlations.

Information about ion–ion correlations may be obtained from different components of conductivity given by eq 4. For this purpose, we distinguish the diagonal terms for which *i* = *j* and the off-diagonal terms with *i* ≠ *j*. The diagonal components are related to diffusion of individual ions, whereas the off-diagonal components describe correlations between motions of different ions. The sum in eq 4 can therefore be partitioned into diagonal and off-diagonal contributions corresponding to three types of ions present in the electrolyte (Li^+^ or EMIM cations and TFSI anions)
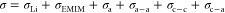
5In the above, σ_Li_, σ_EMIM_, and σ_a_ are the diagonal components describing self-diffusion of Li^+^, EMIM, and TFSI ions, respectively. The other three components are the off-diagonal terms describing cross-correlations between motions of different ions: anion–anion (σ_a–a_), cation–cation (σ_c–c_), and cation–anion (σ_c–a_).

The σ_c–a_ term can be decomposed into parts related to Li-TFSI and EMIM-TFSI correlations
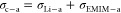
6Analogically, the off-diagonal term σ_c–c_ can be further divided into σ_Li–Li_, σ_Li–EMIM_, and σ_EMIM–EMIM_ contributions
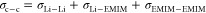
7with σ_X–Y_ describing the correlations between cations of types X and Y.

In the literature, the diagonal terms σ_Me_, σ_EMIM_, and σ_a_ are commonly named “self” contributions, whereas the off-diagonal terms with indices running over the ions of the same type X, σ_Me–Me_, σ_EMIM–EMIM_, and σ_a–a_, are referred to as “distinct” contributions from ion X. A scheme presenting different contributions to the conductivity may be found in the Supporting Information of ref (51).

**Figure 6:**
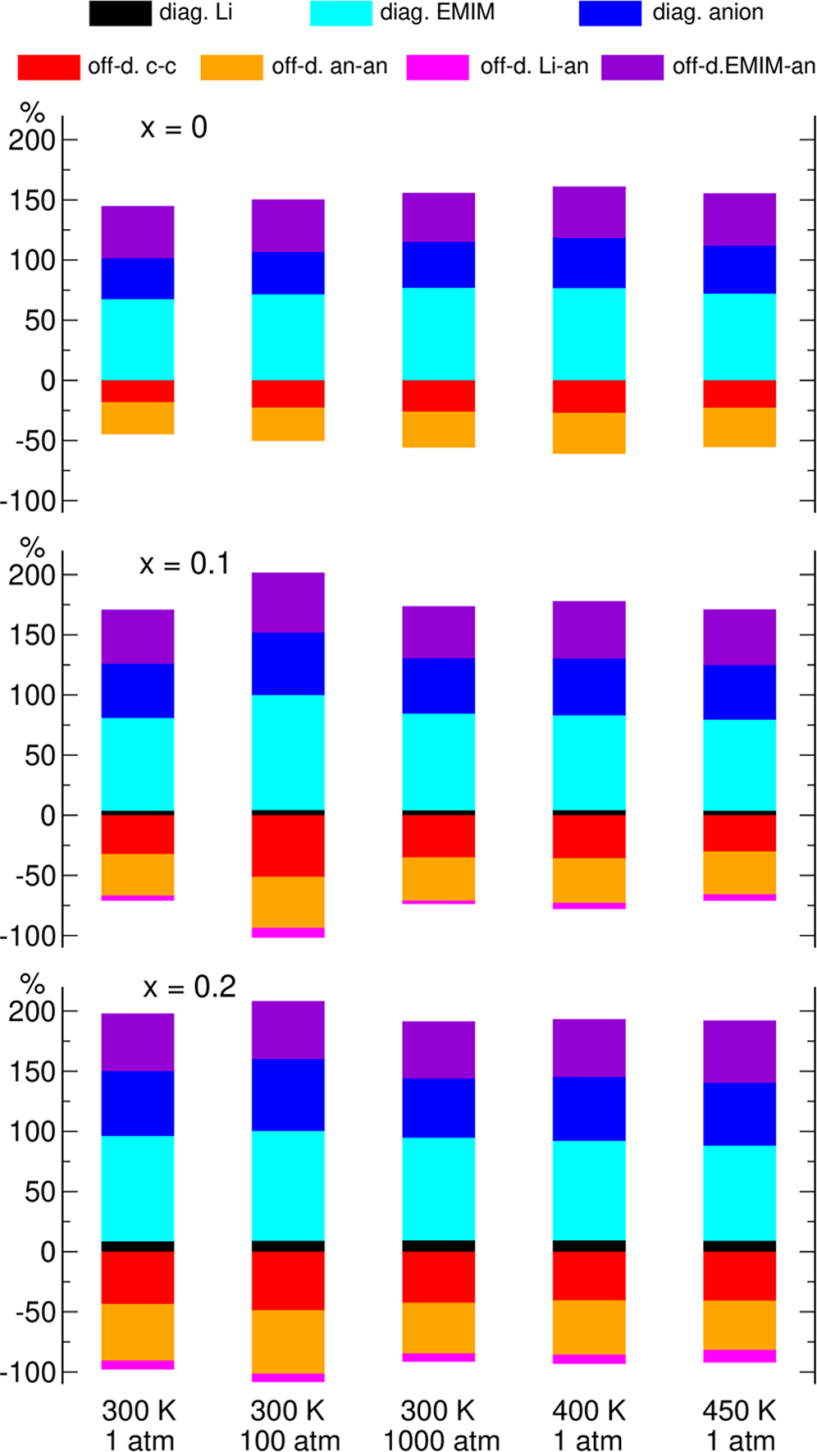
Contributions to the conductivity of the Li_*x*_EMIM_(1–*x*)_TFSI electrolytes. The total conductivity of each system corresponds to 100%.

**Figure 7:**
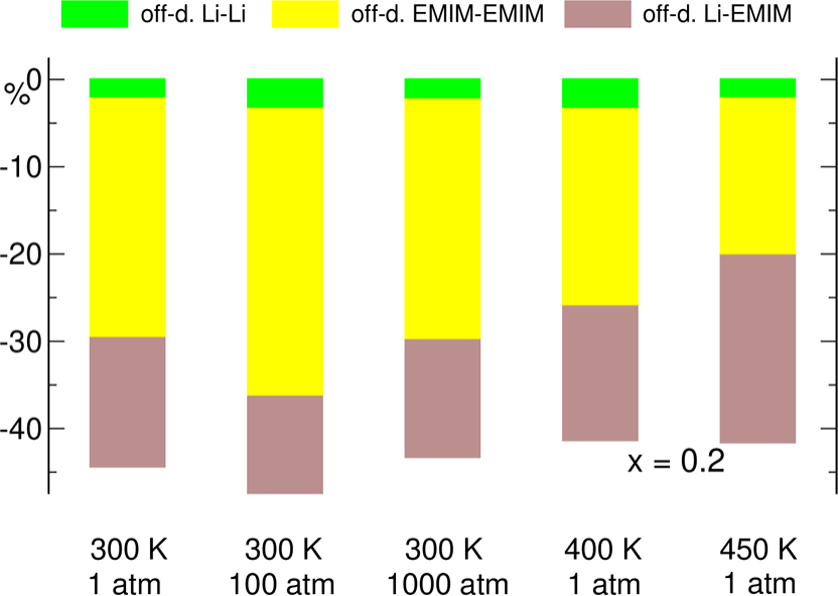
Contributions to the σ_c–c_ component of conductivity for the *x* = 0.2 electrolyte. The total conductivity of each system corresponds to 100%.

Different contributions to conductivity according to eq 5 (with the cation–anion term separated into Li-TFSI and EMIM-TFSI contributions) are shown in Figure 6. For each system separately, the data are rescaled in such a way that 100% corresponds to the conductivity of a given system (the sum of all contributions equals 100%). An alternative scaling, with 100% value corresponding to the total conductivity at standard conditions, is presented in Figure S3 in the Supporting Information. For neat IL (*x* = 0), the σ_c–c_ term consists only of the σ_EMIM–EMIM_ contribution. In other cases (*x* > 0), it can be divided into three components according to eq 7, but for clarity we plotted only the total value of σ_c–c_ in Figure 6. Instead, in Figure 7 (and with the alternative scaling in Figure S4 in the Supporting Information), we presented the contributions to σ_c–c_ for the *x* = 0.2 systems.

Diagonal contributions to the conductivity are the sum of squares; therefore, they are always positive. The self contribution of EMIM cations is larger than that of TFSI anions, owing to the larger diffusion coefficient of the former ions. With increasing LiTFSI content, the self contribution of Li^+^ increases; however, despite the similar diffusion coefficients of Li^+^ and TFSI, the contribution of metal ions is much smaller than the self contribution of anions because of much smaller concentration of Li^+^.

The off-diagonal terms arise from correlations between motions of ions. Both in molecular and ionic liquids, in a typical situation cation–cation and anion–anion motions are anticorrelated; therefore, σ_a–a_ and σ_c–c_ contributions to the conductivity are negative. From Figures 6 and 7, one may see this effect for our electrolytes: distinct contributions as well as cross-terms for Li–EMIM cations are smaller than 0. The case of cation–anion correlations can be more complicated. In molecular liquids, correlated motions of ions with opposite charges lead to negative values of σ_c–a_. On the other hand, momentum conservation imposes anticorrelated movements of cations and anions in ILs and the off-diagonal cation–anion component contributes positively to the total conductivity.^69^ Clearly, this behavior can be observed for the EMIM-TFSI term, but the Li-TFSI contribution is negative, indicating that Li^+^ and anion motions are correlated. The size of this term increases with LiTFSI concentration. Such a feature of metal salt solutions in ILs was experimentally detected as negative transference numbers of metal cations^48^ and confirmed in MD simulations.^49−51^ Nevertheless, the positive σ_EMIM-a_ term dominates over negative σ_Li-a_ and the net effect of cation–anion correlations is a positive contribution to the conductivity. Negative Li-TFSI contribution is consistent with similar diffusion coefficients of both ions, suggesting that the Li^+^ cation and TFSI anions move together in cation–anion aggregates. The *D*_TFSI_ value is larger than *D*_Li_ because all Li^+^ ions are coordinated to anions, whereas even in the *x* = 0.2 electrolyte most TFSI ions are “free”, and therefore *D*_TFSI_ is an average over TFSI coordinated to Li^+^ (slower) and free anions (moving faster).

There is no clear systematic trend noticeable in Figure 6 for changes in temperature or pressure. In the neat IL (*x* = 0), the amount of correlations (the size of off-diagonal terms) is the smallest at 300 K, 1 atm and seems to increase with increasing pressure or temperature, but the changes are rather small. Likewise, for *x* = 0.1 or 0.2, relative contributions to σ for all systems are similar, perhaps with the exception of *p* = 100 atm where the off-diagonal components are larger. As shown earlier, the pressure and temperature can significantly change the residence times. Apparently, the strength of ion–ion correlations does not depend much on the speed of ion exchange in the solvation shell. A similar effect was observed in our previous MD work,^51^ where a change of the metal cation influenced residence times, but not the strength of correlations.

On the other hand, the change of the salt load seems to have larger impact on correlations. Apparently, relative (with respect to diagonal terms) sizes of the off-diagonal components increase with *x*. In particular, for *x* > 0, the negative σ_Li-a_ component appears and there is a substantial increase of the negative cation–cation contribution. The data in Figure 7 suggest that the latter is to a large extent caused by Li–EMIM correlations, which give a larger contribution to σ_c–c_ than could be expected from the 1:4 ratio of Li^+^ to EMIM concentrations.

**Figure 8:**
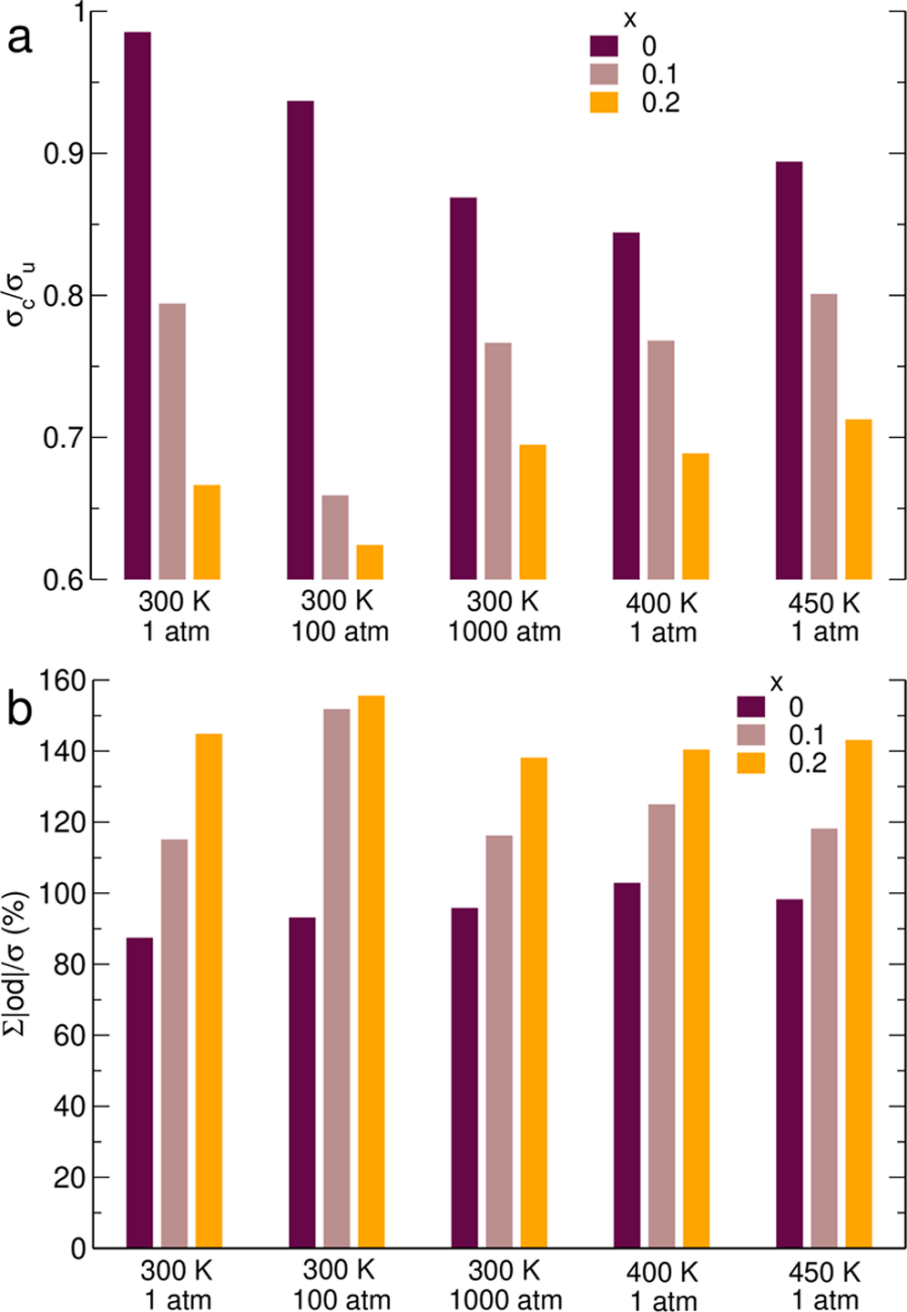
Ratio of correlated-to-uncorrelated conductivities (a) and sum of absolute values of the off-diagonal contributions normalized to the total conductivity (b).

The importance of correlations for ion transport is often assessed by the ratio of correlated conductivity (that is, calculated from the full formula given by eq 4) to the “uncorrelated” value σ_u_, retaining only diagonal terms in eqs 4, and 5, in which case the conductivity is proportional to the mean value of diffusion coefficients of ions (weighted by their concentrations). Without correlations, σ_c_ = σ_u_; therefore, the deviation of the σ_c_/σ_u_ ratio from unity is often considered as a measure of the degree of correlations in the electrolyte. We plotted values of σ_c_/σ_u_ in Figure 8a. Additionally, in Figure 8b we also displayed the sum of absolute values of the off-diagonal contributions normalized to the total conductivity.

The data for neat IL (*x* = 0) at 300 K, 1 atm show why the σ_c_/σ_u_ ratio is not sufficient to assess the strength of correlations in ionic liquids. The off-diagonal contributions reach more than 80% of the total conductivity; however, the σ_c_/σ_u_ ratio is 0.986 because the positive off-diagonal σ_EMIM-a_ contribution almost exactly cancels negative σ_a–a_ and σ_c–c_ components (Figure 6). In typical salt solutions in molecular liquids, all off-diagonal components are negative; therefore, it is safe to assume that the value of σ_c_/σ_u_ close to 1 implies that correlations are small. This is not true in ILs, where the off-diagonal components have different signs, and their net effect may be close to 0 even in the case of strong correlations in the electrolyte.

It follows from Figure 8b that correlations are important in all studied electrolytes, and the sum of absolute values of off-diagonal contributions is between 80 and 150% of the total conductivity. There are differences between systems simulated under different conditions; however, it is clear that the salt concentration has larger impact on correlations than the temperature or pressure. The total size of correlations increases approximately linearly with the salt fraction *x* (except the system at 300 K, 100 atm). Accordingly, the ratio of correlated-to-uncorrelated conductivity decreases systematically with salt load (Figure 8a). It is consistent with the computational findings that when the salt concentration in IL increases the major factor responsible for the decrease of conductivity is not the decrease of diffusion coefficients (resulting from increased viscosity) but the increase of destructive correlations.^43,51^

Transference numbers are commonly used to quantify the contributions of different ions to the conductivity of the system. Therefore, we calculated values of transference numbers of all ions from the conductivity partitioning given by eqs 5–7; e.g., the Li^+^ transference number
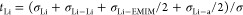
8and using analogous expressions for *t*_EMIM_ and *t*_a_.

For the neat EMIM-TFSI liquid, transference numbers of the IL ions are the same regardless of the temperature or pressure (changes are smaller than 0.005) and amount to 0.7 and 0.3 for EMIM and TFSI ions, respectively. The cations of the IL are more mobile than the anions and contribute to a greater extent to the charge transport. In LiTFSI solutions, *t*_TFSI_ is between 0.28 and 0.32; lower values are observed for *x* = 0.2. Transference number of EMIM cations increases in salt solutions up to 0.76. Our values of *t*_EMIM_ and *t*_TFSI_ differ by about 0.1 from the experimental results;^48^ the difference is smaller than the cumulated uncertainty of measurement and simulations.

**Figure 9:**
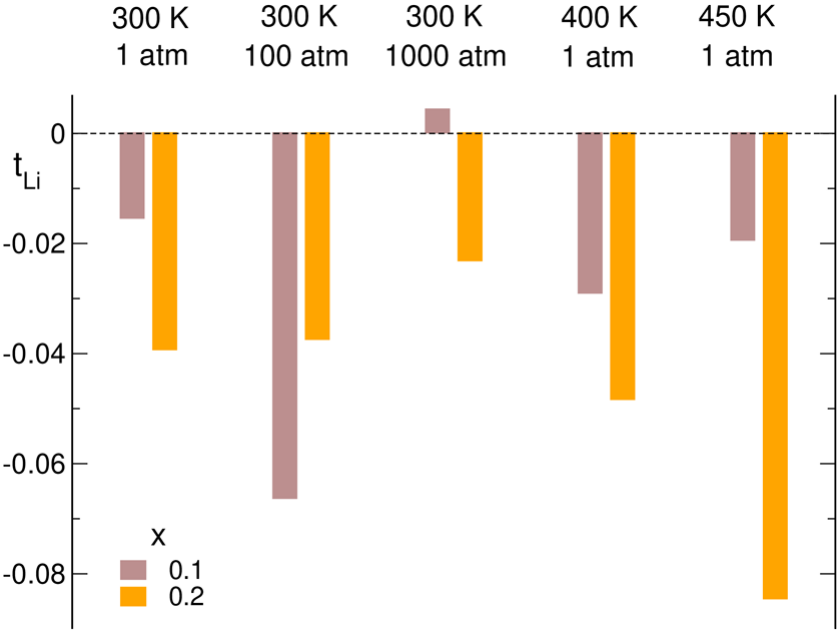
Estimated Li^+^ transference numbers.

The most interesting in the context of recent experimental and theoretical works^48−51^ are the values of *t*_Li_, presented in Figure 9. One should note that these values result from the delicate balance of smallest contributions shown in Figures 6and 7, mostly off-diagonal terms with relatively large uncertainties, and therefore *t*_Li_ values are prone to large error bars. There is no apparent trend visible in Figure 9, but almost all values of *t*_Li_ are negative between −0.015 and −0.08. The sole exception is the small positive value of 0.004 obtained for the system *x* = 0.1 at 300 K, 1000 atm. In this case, however, the uncertainty of *t*_Li_ is expected to be the largest because of low salt content and small mobility of ions at high pressure; therefore, we attribute positive *t*_Li_ for this system to the large error bar. The transference numbers for the two types of cations, *t*_EMIM_ and *t*_Li_, are anticorrelated: the more negative *t*_Li_, the larger the *t*_EMIM_ value. Our estimates of *t*_Li_ between −0.015 and −0.04 obtained at 300 K, 1 atm agree well with experimental values of effective transference numbers between −0.02 and −0.04.^48^

We applied the approach presented in ref (50) to estimate the cation effective charges *q*_eff_ through comparing the Li^+^ uncorrelated conductivities to the transference numbers calculated above from the full analysis of correlations in the electrolyte. The relative errors are large for the same reasons as in the case of *t*_Li_; therefore, we did not observe any clear trend of changes and the average *q*_eff_ are between −0.8 and −0.9. Generally, the values of *q*_eff_ are expected to be greater than 1 – *N*, where *N* is the number of anions in the solvation shell of the Li^+^ ion. The estimated values agree therefore with the most probable solvation of the lithium cation by two TFSI anions leading to the formation of [Li(TFSI)_2_]^−^ complexes, as predicted from the CNs in Section 3.1. These findings are in agreement with the conclusions of the experimental work^48^ that the effective charge of the Li ion solvated in EMIM-TFSI is −1.

## 4 Conclusions

We performed classical MD simulations with a polarizable force field for LiTFSI solutions in EMIM-TFSI IL. Three salt concentrations were used, and we changed the temperature and pressure applied in the simulations. Although the temperature and pressure change the density of the electrolyte, the local structure of the Li^+^ solvation shell and coordination numbers are only weakly affected by the conditions.

All systems exhibit a large degree of ion–ion correlations amounting to 80–150% of the total conductivity. However, as typical for ionic liquids, anticorrelations between anions and cations of the IL contribute positively to the conductivity and cancel a large part of destructive cation–cation and anion–anion correlations. On the other hand, in all cases, motions of Li^+^ cations and TFSI anions are correlated, contributing toward decrease of conductivity and leading to the phenomenon of negative Li^+^ transference numbers.

Changes in the temperature or pressure affect the diffusion coefficients and conductivity of the electrolyte. However, they have a rather limited effect on the degree of correlations in the IL. The impact of salt concentration is much larger—correlations increase with increasing salt content and cross-correlations reduce the overall conductivity. Nevertheless, the correlations between motions of IL anions and metal cations persist for all values of temperature or pressure, even though the conditions significantly change the timescale of anion exchange in the Li^+^ coordination shell. As a result, negative transference numbers of metal cations are observed regardless of the pressure or temperature.

Although we used only one salt/IL pair as a model system, other MD studies on conductivity and ion transport in ILs^43,49−51,70^ suggest that the effects of correlations are similar in all electrolytes of this kind; therefore, the findings reported here could be probably generalized to other ILs.

## Supporting Information Available

Sizes of the simulation boxes, information on pressure fluctuations, and plots applying alternative scaling of conductivity contributions (PDF)
