# Supplementary material for: How Temperature, Pressure, and Salt Concentration Affect Correlations in LiTFSI/EMIM-TFSI Electrolytes: A Molecular Dynamics Study
Source: J Phys Chem B. 2021 Oct 28;125(44):12292–302. doi: 10.1021/acs.jpcb.1c07782 (PMC8591607; doi:10.1021/acs.jpcb.1c07782)
Supplement: Supplementary file 1 — jp1c07782_si_001.pdf [file jp1c07782_si_001.pdf]

**How Temperature, Pressure and Salt Concentration Affect the Correlations in LiTFSI/EMIM-TFSI Electrolyte: A Molecular Dynamics Study**

Piotr Kubisiak, Piotr Wróbel, Andrzej Eilmes*

*Faculty of Chemistry, Jagiellonian University, Gronostajowa 2, 30-387 Kraków, Poland*

# Supporting Information

* e-mail: eilmes@chemia.uj.edu.pl

Table S1. Linear Sizes (Å) of Simulation Boxes under Different Conditions.

| system | 300 K, 1 atm | 300 K, 100 atm | 300 K, 1000 atm | 400 K, 1 atm | 450 K, 1 atm |
|---|---|---|---|---|---|
| $x$ = 0: 142 EMIM-TFSI | 39.365 | 39.247 | 38.536 | 40.622 | 41.269 |
| $x$ = 0.1: 15 LiTFSI + 135 EMIM-TFSI | 39.689 | 39.553 | 38.778 | 40.983 | 41.658 |
| $x$ = 0.2: 34 LiTFSI + 136 EMIM-TFSI | 40.958 | 40.804 | 39.931 | 42.335 | 43.050 |

Pressure fluctuations in MD simulations

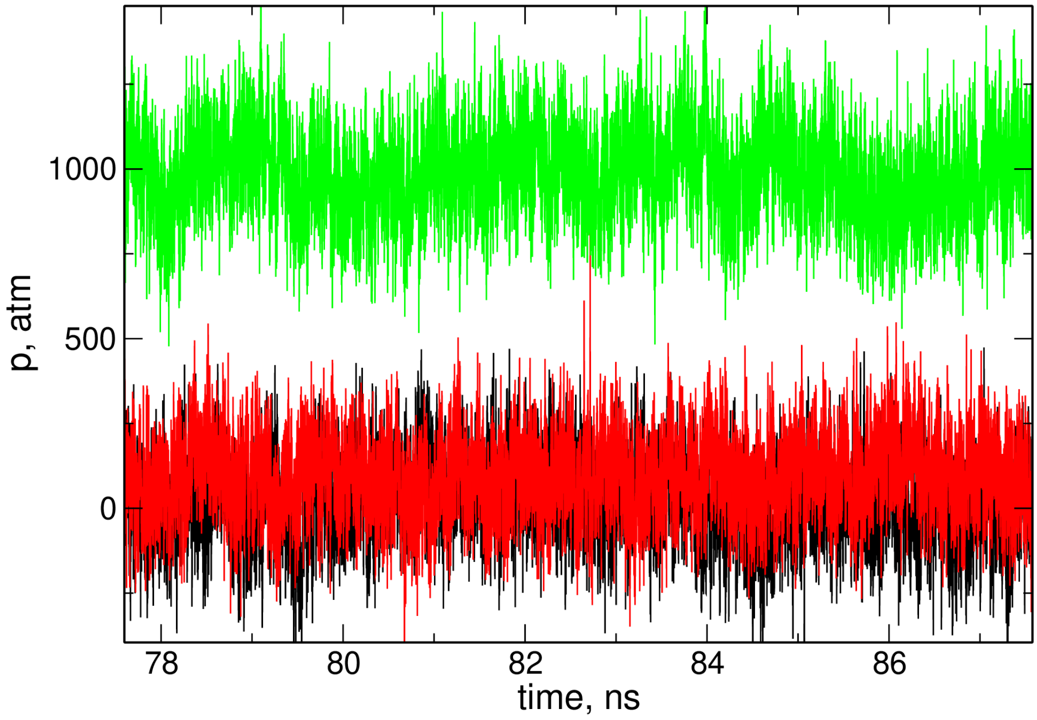


Figure S1. Fluctuations of the pressure observed for the *x* = 0 system simulated at 300 K and 1 atm (black), 100 atm (red) or 1000 atm (green). Values are averages over 1 ps time intervals (2000 MD steps) as given in the MD output files.

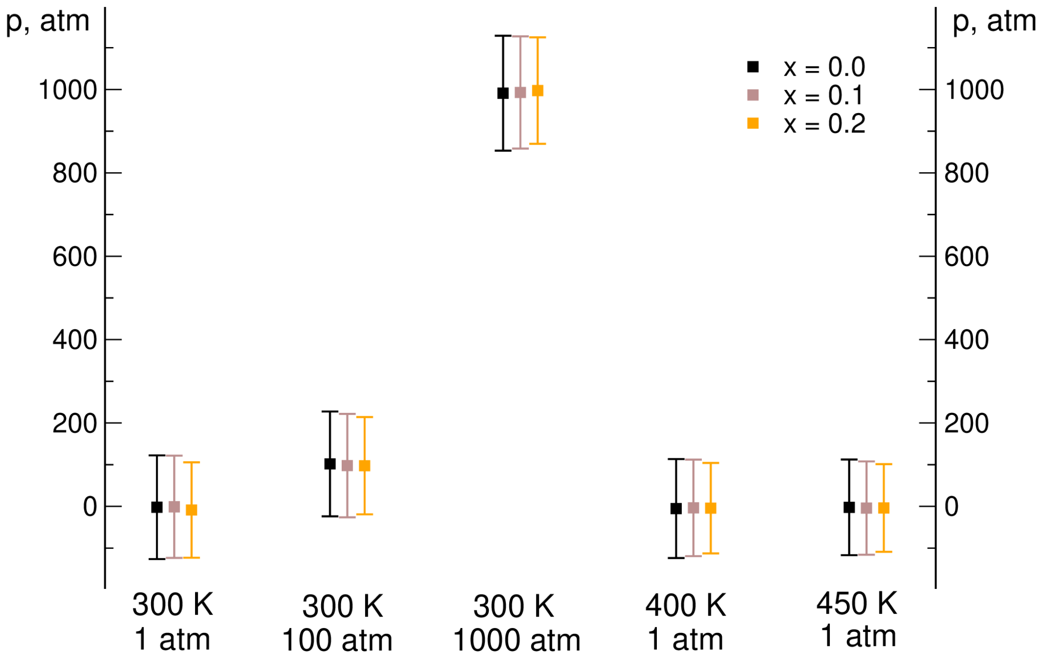


Figure S2. Average values and standard deviations of pressure recorded during MD simulations for different systems and conditions.

Pressure fluctuations only slightly increase in the systems simulated at p = 1000 atm. There is a systematic trend of decreasing pressure fluctuations with increasing salt concentration.

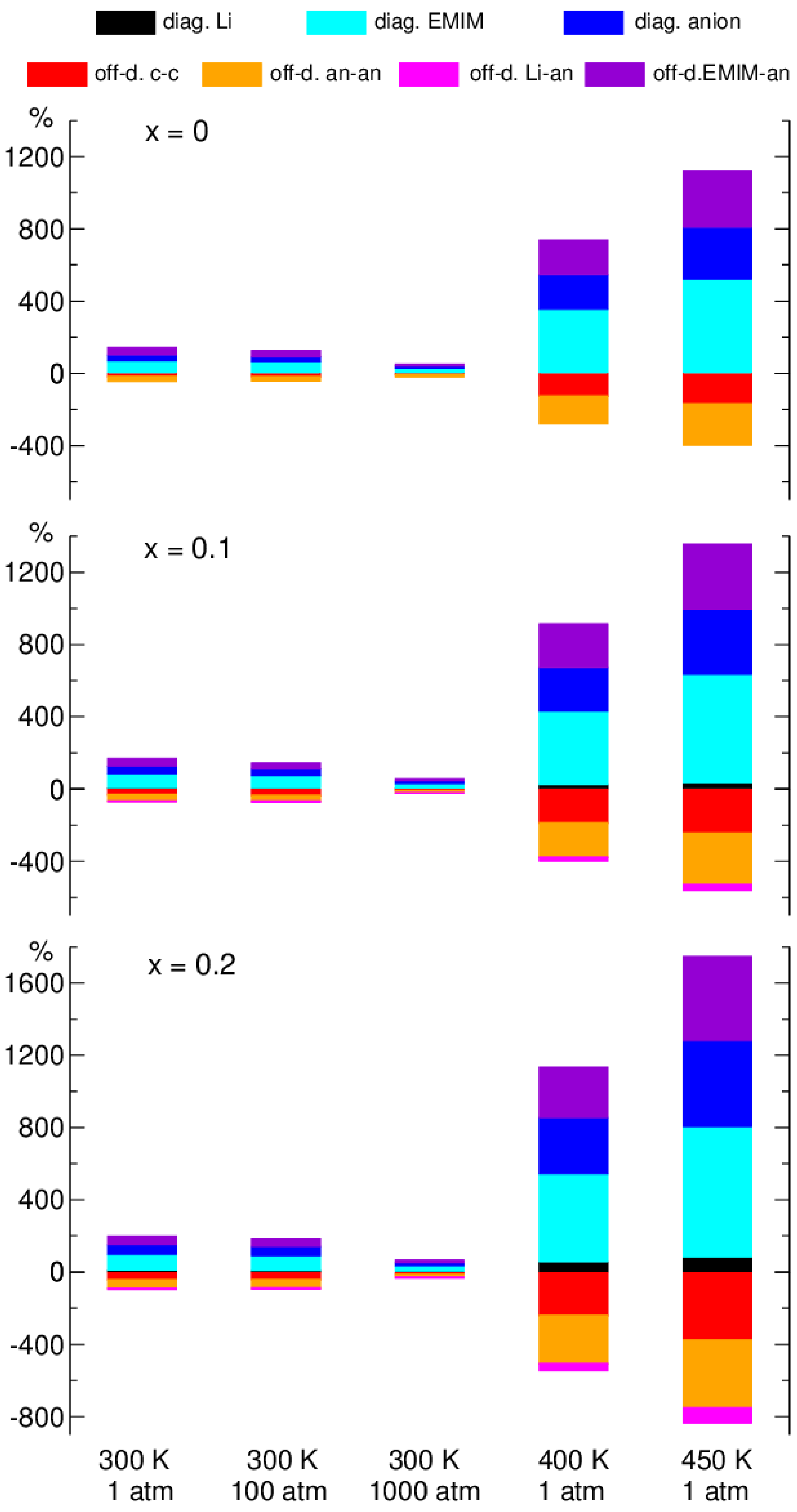


Figure S3. Contributions to the conductivity of the $Li_xEMIM_{(1-x)}TFSI$ electrolytes. For each concentration *x*, the total conductivity at 300 K, 1 atm corresponds to 100 %.

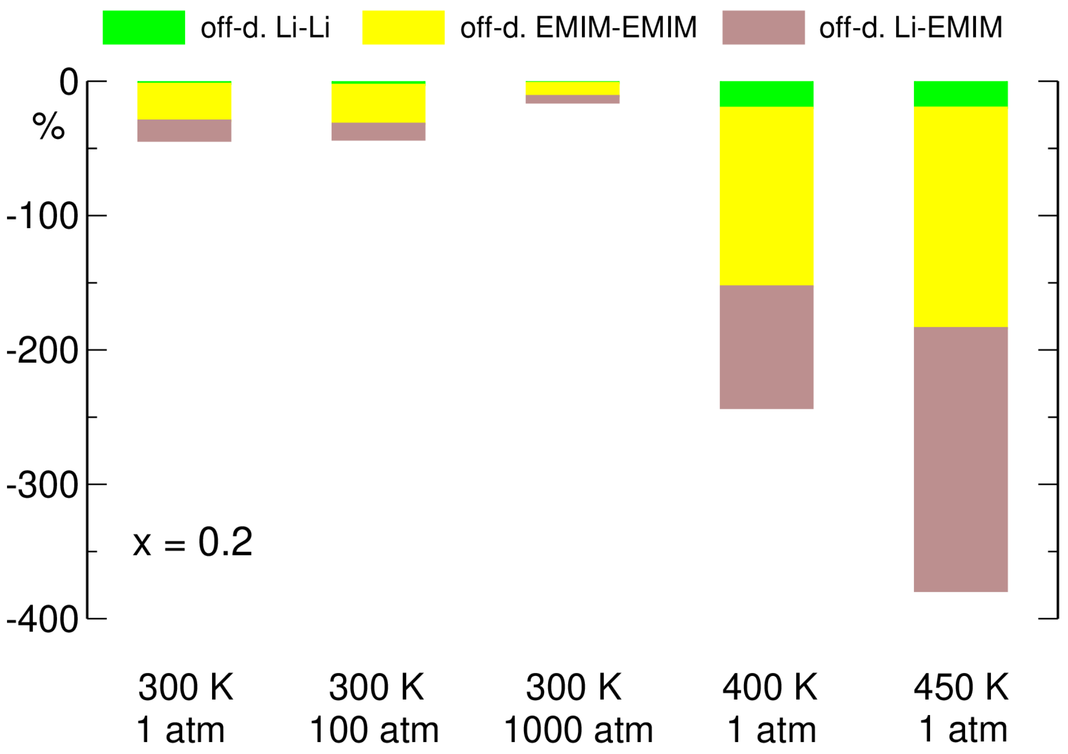


Figure S4. Contributions to the $\sigma_{c\text{-}c}$ component of the conductivity for the $x$ = 0.2 electrolyte. The total conductivity at 300 K, 1 atm corresponds to 100 %.
